# Reduction of artifacts associated with missing data in coherent diffractive imaging

**DOI:** 10.1107/S1600577524010956

**Published:** 2025-01-01

**Authors:** Erik Malm, Yuriy Chushkin

**Affiliations:** a MAX IV Laboratory Lund University 22100Lund Sweden; b ESRF – The European Synchrotron, 71 Avenue des Martyrs, 38000Grenoble, France; Paul Scherrer Institute, Switzerland

**Keywords:** phase retrieval, coherent diffractive imaging, tomography

## Abstract

Coherent diffractive imaging experiments often collect incomplete datasets containing regions that lack any measurements. These regions can arise because of beamstops, gaps between detectors, or, in tomography experiments, a missing wedge of data due to a limited sample rotation range. We describe practical and effective approaches to mitigate reconstruction artifacts by bringing uniqueness back to the phase retrieval problem. This is accomplished by looking for a solution that both matches the data and has minimum total variation, which essentially sets the unconstrained modes to reduce oscillations within the reconstruction. Two algorithms are described. The first algorithm assumes that there is an accurate estimate of the phase and can be used for pre- and post-processing. The second algorithm attempts to simultaneously minimize the total variation and recover the phase. We demonstrate the utility of these algorithms with numerical simulations and, experimentally, on a large, three-dimensional dataset.

## Abstract 2

We describe practical approaches to mitigate reconstruction artifacts in coherent diffractive imaging experiments which stem from regions that lack data. The utility of these approaches is demonstrated with 3D numerical simulations and, experimentally, on a coherent diffractive imaging dataset.

## 1. Introduction

X-ray coherent diffractive imaging (CDI) is a powerful method for imaging micrometre-sized samples in two and three dimensions. The lack of image-forming optics makes it well suited for the X-ray regime where optics are difficult and expensive to fabricate. Instead of using lenses, CDI obtains real-space images via numerical iterative phase retrieval algorithms (Gerchberg & Saxton, 1972 ▸; Fienup, 1978 ▸; Miao *et al.*, 1998 ▸; Chapman & Nugent, 2010 ▸; Quiney, 2010 ▸; Shechtman *et al.*, 2015 ▸). Combined with the ability, at least in principle, to obtain wavelength-limited resolution, it can be used to image nanometre-scale features in three dimensions (3D). Unfortunately, the diffraction signal falls off rapidly as a function of the distance from the center of the scattering pattern. Due to the limited dynamic range of detectors, a large beamstop blocking the intense direct beam must be used to collect high-frequency data. Beamstops larger than a few speckles lead to unconstrained modes that cannot be uniquely determined from the data (Thibault *et al.*, 2006 ▸). Thibault *et al.* (2006 ▸) defined unconstrained modes as Hermite–Gaussian modes that cannot be determined by the support-data pair. A limited sample rotation range in a tomography experiment below 180° results in a wedge of missing data. In addition, the finite angular step size of the sample rotation leads to regions of missing data that become larger for higher frequencies. Some X-ray detectors comprise multiple chips that contain gaps. These experimental realities often lead to datasets with significant amounts of missing data. In such conditions, conventional phase retrieval algorithms often provide reconstructions with severe artifacts which obscure the sample’s features. For this reason, it is important to be able to mitigate these artifacts in a controlled and practical way.

Here, we solve this problem by looking for a solution with minimum total variation (TV) which still satisfies the measurements. The approaches described here are most similar to those described by Chartrand (2007 ▸) and He *et al.* (2015 ▸). Other, more recent, approaches are based on neural networks (Bellisario *et al.*, 2022 ▸) and TV with regularization (Yokoyama *et al.*, 2022 ▸). Unlike the majority of TV methods, our aim is not to regularize the solution but only remove reconstruction artifacts by bringing uniqueness back to the phase retrieval problem. In this way, we remove the ambiguity related to specifying arbitrary regularization constants needed to balance data fidelity and regularization terms.

Expanding on the work of He *et al.* (2015 ▸), we explore the performance of these methods in 3D and with missing high-frequency data due to gaps in the detector, a missing wedge and smaller wedge-like regions which result from the finite step size of the sample rotation. We show the impact of these methods on how reliably the phase is recovered through the phase retrieval transfer function (PRTF) values (Chapman *et al.*, 2006 ▸). In addition, the stark differences between the recovered signal in Fourier space are visualized. Finally, we find that, by iterating between TV and phase retrieval steps, further improvements to the reconstruction quality can be made. The simplicity of the algorithms described here means that they can be applied to large 3D experimental datasets.

## 2. Phase retrieval theory

### 2.1. Problem formulation and algorithm

In this section we describe two approaches for handling missing data in a CDI experiment. In the first problem we assume that the measurements have an estimate of the phase. The aim is to minimize TV(*u*) = *∥*∇*u∥*_1_ while still satisfying the Fourier measurements. Mathematically, we aim to solve 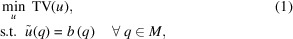
where 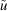 = 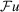 denotes the Fourier transform of *u*, and *M* is the region of the measurement domain where signal has been measured. In our context, *u* is proportional to the electron density; however, in a 2D transmission CDI experiment it is proportional to the X-ray exit surface wave. We will refer to *u* as the reconstruction and note that the exact interpretation depends on the specific forward model associated with the experiment. In problem (1), *b* is complex-valued and already has an estimate of the phase. This is applicable for in-painting a dataset or post-processing a reconstruction obtained through either phase retrieval or some holographic approach (Malm, 2021 ▸). The precise amount of phase necessary to recover an accurate reconstruction is not studied here, but could be the subject of future studies.

Next, consider the problem where the measurements contain information about the magnitude of the signal, but completely lack any phase information. Now the problem becomes 
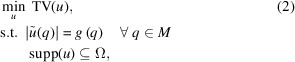
where *g* are the real, positive amplitude measurements taken over the measurement domain, *M*. Here, we have included the support constraint stating that there is a region, Ω, where *u* is allowed to be non-zero. Additional constraints can be included if there exists information which can further constrain the solution. Now, the problem is to minimize the TV cost function while recovering the phase using the typical modulus and support constraints.

### 2.2. Algorithm

We describe two algorithms for solving problems (1) and (2). The first algorithm, which we call ‘TV’, is suitable for inpainting an incomplete dataset or post-processing a reconstruction. The second algorithm, which we refer to as ‘TVRAAR’, attempts to perform phase retrieval while minimizing the TV of the reconstruction. The phase retrieval algorithm we use is relaxed averaged alternating reflections (RAAR) (Luke, 2005 ▸) as it appears to be the most stable and robust with respect to noise.

#### 2.2.1. TV algorithm

We use a simple projected gradient descent approach to solve problem (1). That is, gradient descent is used to minimize TV(*u*) while projecting onto the measurements in Fourier space. The update for the *k* + 1 iteration is then 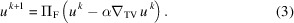
Above, α is the step size and ∇_TV_ denotes the gradient of the TV cost function with respect to *u*. Mathematically, ∇_TV_(*u*) = 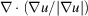, where |…| denotes the vector magnitude at each point in space. The Fourier projection is defined by 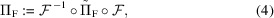
where the projection in Fourier space is 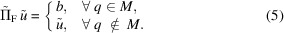

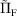
 simply replaces the values in the Fourier domain with the measured values, *b*, in the regions containing data. Equation (3) is applied repeatedly for a fixed number of iterations. To ensure the solution is not regularized, the value of α is decreased to some minimal value at the end.

#### 2.2.2. TVRAAR algorithm

In this section, phase retrieval is performed during the TV minimization. To accomplish this, we simply inject a gradient descent step into the RAAR algorithm (Luke, 2005 ▸) to push the solution towards lower TV values. The TVRAAR algorithm becomes 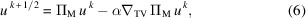

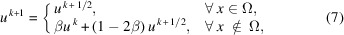
where the modulus projection is given by 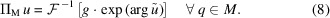
The modulus projection replaces the Fourier amplitude of our estimate with the phaseless measurements. Ω: = {*x* ∈ *D* | *u*(*x*) ≠ 0} is an estimate of the support of *u*, and β is a real constant fixed to 0.8 during the reconstructions.

## 3. Numerical simulations

**Figure 1:**
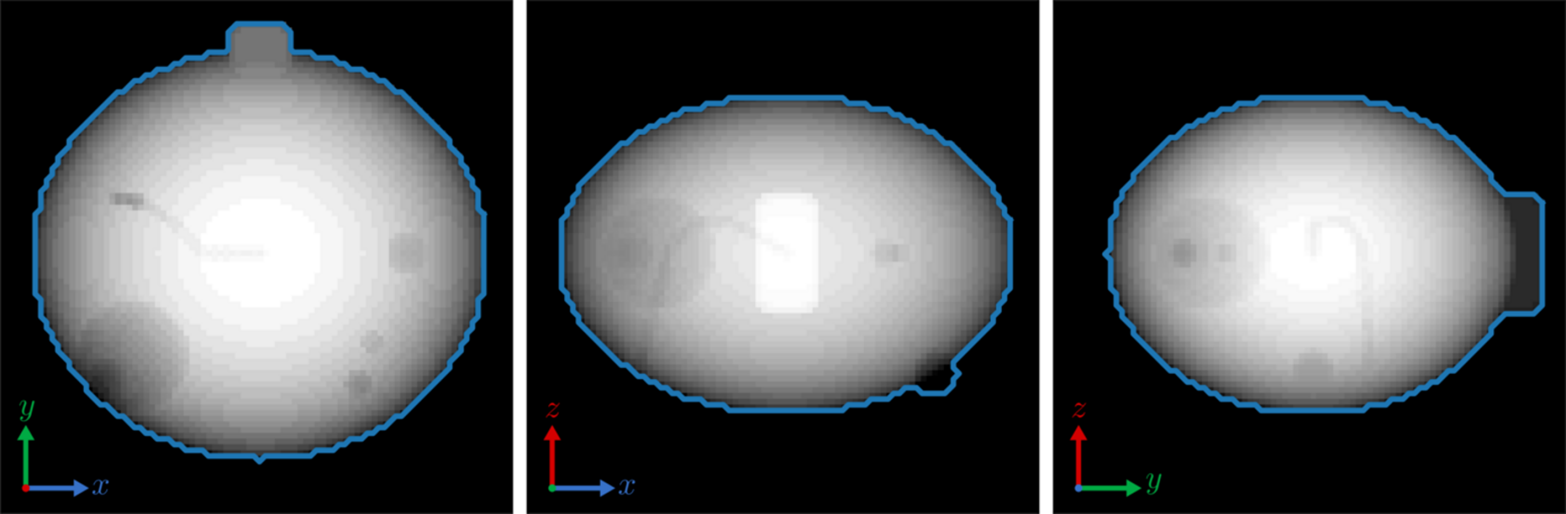
Projections of the particle’s 3D electron density along different directions shown on a linear scale. The outline of the fixed support is shown by the blue lines.

In this section, results from several numerical simulations are presented which illustrate the performance of three different algorithms. To accomplish this, 3D datasets are simulated with different beamstop diameters, missing wedge angles (θ) and signal-to-noise ratios (SNRs). A 3D ellipsoidal sample was created containing features one might expect from micrometre-sized particles in a 3D CDI experiment. These features include several regions with different electron densities, a crack-like feature through the center, and small spherical voids. Fig. 1 ▸ shows three orthogonal projections of the particle used for these simulations.

The ideal intensities were simulated by applying a Fourier transform to the sample, multiplying by the 3D beamstop and missing wedge masks and computing the intensity by squaring the magnitudes. To calculate the measured noisy intensities, Gaussian white noise was added with standard deviation σ and the values were rounded to the nearest integer value. A spherical beamstop was used as this is the shape a circular beamstop would produce experimentally after the 2D frames have been assembled into the 3D dataset. Unless used as the variable, σ and θ were fixed to 50 counts and 10°, respectively, while the beamstop diameter was set to approximately four speckles in each direction. Here, the SNR is defined as SNR = 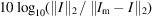, where *I* and *I*_m_ are the ideal and measured intensities respectively. The l_2_-norm is denoted by ∥…∥_2_ which is calculated only in regions with measurements (*M*).

Three algorithms are used for comparison. The first, ERHIO, is a conventional phase retrieval algorithm that utilizes a combination of error reduction (ER) and hybrid input–output (HIO) (Fienup, 1982 ▸) updates. The second, TV–ERHIO, uses the TV algorithm after ERHIO as a post-processing step. The third, TVRAAR–ERHIO, uses a combination of TV and TVRAAR algorithms after ERHIO. Details about these algorithms are provided in Appendix *A*[App appa]. A fixed support was used which was approximately one pixel larger on all sides than the exact support. Outlines of the support are shown by the blue lines in Fig. 1 ▸. To avoid imaginary values in the amplitude measurements, intensity values below zero were set to zero before applying the square root. The final reconstruction for each algorithm is an average of ten independent reconstructions. The error metric used to determine the quality of each reconstruction is a direct comparison between the reconstruction *u* with the simulated sample *u*_0_. It is defined by *E* = ∥|*u*| − |*u*_0_|∥_2_ / ∥*u*_0_∥_2_. We use the difference in amplitudes in *E* because *u*_0_ is real and positive and it removes the need to determine a phase rotation to match *u* to *u*_0_.

**Figure 2:**
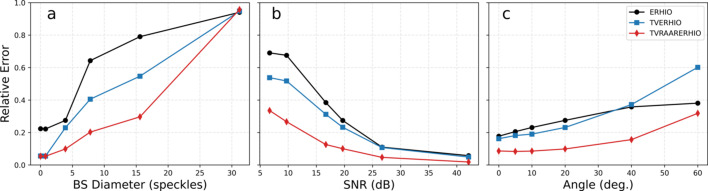
Relative errors of each algorithm while varying (*a*) beamstop diameter, (*b*) SNR and (*c*) missing wedge angle.

Fig. 2 ▸ shows plots of the relative errors for each of the three algorithms for different beamstop diameters, SNR and missing wedge angle values. Both TV algorithms out-perform the ERHIO algorithm during essentially the entire range of parameters. In the extreme cases, such as beamstop diameters of 15 speckles, low SNR values and θ = 60°, TVRAAR still shows a significant improvement. Reconstructions and error evolution for a few of these datasets are shown in the figures in the supporting information. When the beamstop diameter approaches 30 speckles, essentially all the algorithms fail to recover a meaningful reconstruction. However, the TVRAAR algorithm has approximately the same error at a beamstop size of 10 to 15 speckles as ERHIO has with essentially no beamstop, showing the significant robustness of this method to missing data. This fact and the reconstructions provided in the supporting information suggest that TVRAAR may also help with phase retrieval stagnation issues that occur when the support constraint cannot distinguish between *u*(*x*) and *u*(−*x*) (Fienup & Wackerman, 1986 ▸). All the plots illustrate the robustness of the TV algorithms to noise and missing data.

The experimental dataset described in the next section has a missing wedge angle of 28°. This dataset contains regions of missing data that arise from gaps between detectors, missing wedge, beamstop and high-frequency missing data due to the finite rotational step size of the sample. This makes it difficult to draw a direct comparison between the beamstop diameter values in the simulations and the experimental dataset. Nevertheless, the next section illustrates the robustness of TVRAAR to these experimental difficulties.

## 4. Experiment and data analysis

CDI measurements were performed at the ID10 beamline (Chushkin *et al.*, 2014 ▸). A coherent X-ray beam of 8.09 keV radiation was selected by a Si(111) channel-cut monochromator. The beam size used was 10 µm × 10 µm with intensity of 3.9 × 10^11^ photons s^−1^. Coherent diffraction patterns were measured by an Eiger2 4M CdTe detector placed 5.45 m downstream from the sample. As a sample, we used CaCO_3_ microparticles deposited on a 100 nm-thick silicon nitride membrane. A set of 2D diffraction patterns of 3 s exposure were collected by rotating the sample from −74° to 78° with 0.25° angular step.

The 2D diffraction datasets were assembled into a 3D diffraction volume with a final shape of 650^3^ pixels. Our phase retrieval procedures depend on having a good estimate of the sample support. This was determined with a preliminary phase retrieval procedure using a semi-automatic shrinkwrap technique (Marchesini *et al.*, 2003 ▸). The phase retrieval procedures used this fixed support to obtain the final reconstruction. Phase retrieval was performed using either ERHIO or TVRAAR–ERHIO as described earlier in Section 3. For brevity we describe the algorithm as ‘TVRAAR–ERHIO’, but the procedure includes both TV and TVRAAR algorithms. The TV algorithms start with a relatively large step size, α, which was determined heuristically and towards the end reduced to a minimum value to prevent regularization of the solution. The exact algorithm is described in Appendix *A*[App appa]. As a final step, 40 reconstructions were aligned (Guizar-Sicairos *et al.*, 2008 ▸) and averaged to obtain the final 3D images.

## 5. Results and discussion

Here, we compare the reconstructions obtained with conventional phase retrieval algorithms, ERHIO, and phase retrieval combined with TV refinement, TVRAAR–ERHIO, described earlier. The TVRAAR–ERHIO algorithm uses a combination of ER, HIO, TV and TVRAAR algorithms. The full details of this algorithm are provided in Appendix *A*[App appa].

**Figure 3:**
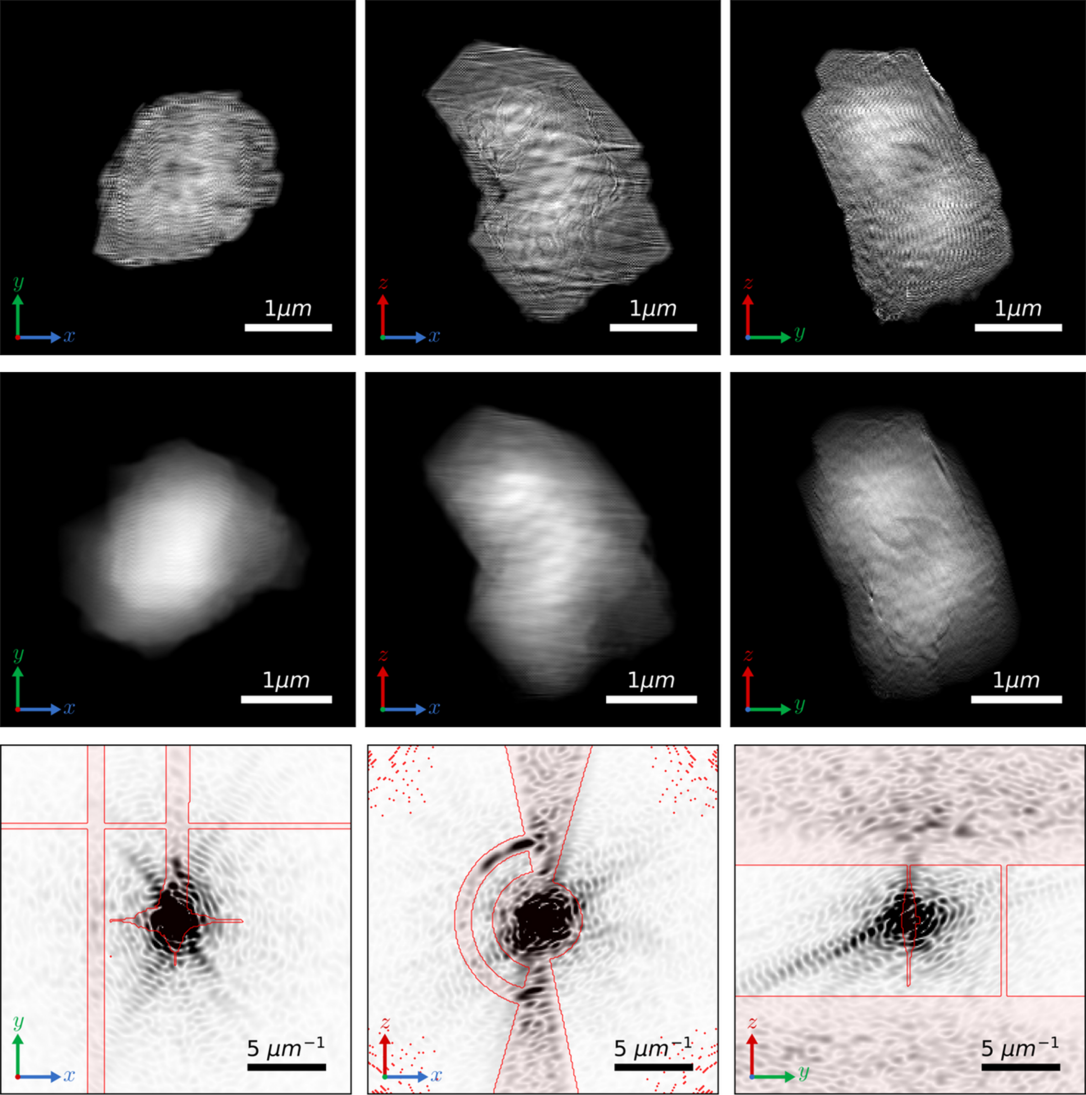
Three-dimensional reconstruction obtained with the ERHIO algorithm. Top row: orthogonal slices through the 3D electron density. Middle row: orthogonal projections of the reconstruction. Bottom row: slices through the reconstruction in Fourier space with the masked regions shaded red. The highest intensities have been clipped to help visualize the lower values. Note that a reversed gray-scale colormap is used in the bottom row so that the smallest values correspond to white. All images are shown on a linear scale.

Figs. 3 ▸ and 4 ▸ show the reconstructions of the particle using ERHIO and TVRAAR–ERHIO, respectively. Orthogonal slices through the 3D sample are shown in the top row, while projections along three orthogonal directions are shown in the middle row. The bottom rows show orthogonal slices through one out of the 40 reconstructions in the Fourier domain. For visualization purposes, the highest values were clipped and only the central 250 × 250 pixels are shown. The red lines indicate the boundary of the mask which separates regions with and without data. The first column shows a vertical region of missing data due to the separation between individual detector modules. The missing data in the middle of the image is due to a beamstop and a cross region where the background could not be reliably subtracted. The middle column shows the missing wedge due to the limited angular range of the measurements.

We can clearly see artifacts in the ERHIO reconstruction which appear mostly as high-frequency oscillations. These artifacts obscure the features in the sample which become visible in the TVRAAR–ERHIO reconstruction. Indeed, if we look at the Fourier space in Fig. 3 ▸ (bottom row) we see that the values in the missing data regions appear too large compared with the surrounding measurements. These values are not constrained by the phase retrieval constraints and result in the rapid fluctuations in the electron density. In contrast, the TV refinement has removed these artifacts resulting in a signal in Fourier space which matches the surrounding measurements. Various features in the sample are now visible such as small voids and regions with different electron densities.

The reliability of the reconstruction procedure is described by the PRTF values, given by 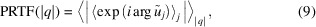
where |*q*| is the radial distance from the center, 〈…〉_*j*_ indicates the average over the 40 independent reconstructions, and 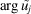 is the phase of the *j*th reconstruction in the Fourier domain. 〈…〉_|*q*|_ indicates an average over shells of constant |*q*|. The PRTF value is averaged over shells of constant |*q*| and is a function of radial distance from the center of the diffraction pattern. Intuitively, if the recovered phases are inconsistently recovered then a random walk in the complex plane occurs resulting in a final vector that lies near the origin, and, consequently, a small PRTF value. If, on the other hand, the phases are all the same, then the vectors point in the same direction and the value will lie close to 1.

The PRTF values associated with the two reconstructions from Figs. 3 ▸ and 4 ▸ are shown in Fig. 5 ▸. We clearly see a significant improvement with the TV refinement procedure. In particular, the shoulder, where the values begin to drop, changes from approximately 70 nm to approximately 20 nm, suggesting a significant improvement in resolution.

**Figure 4:**
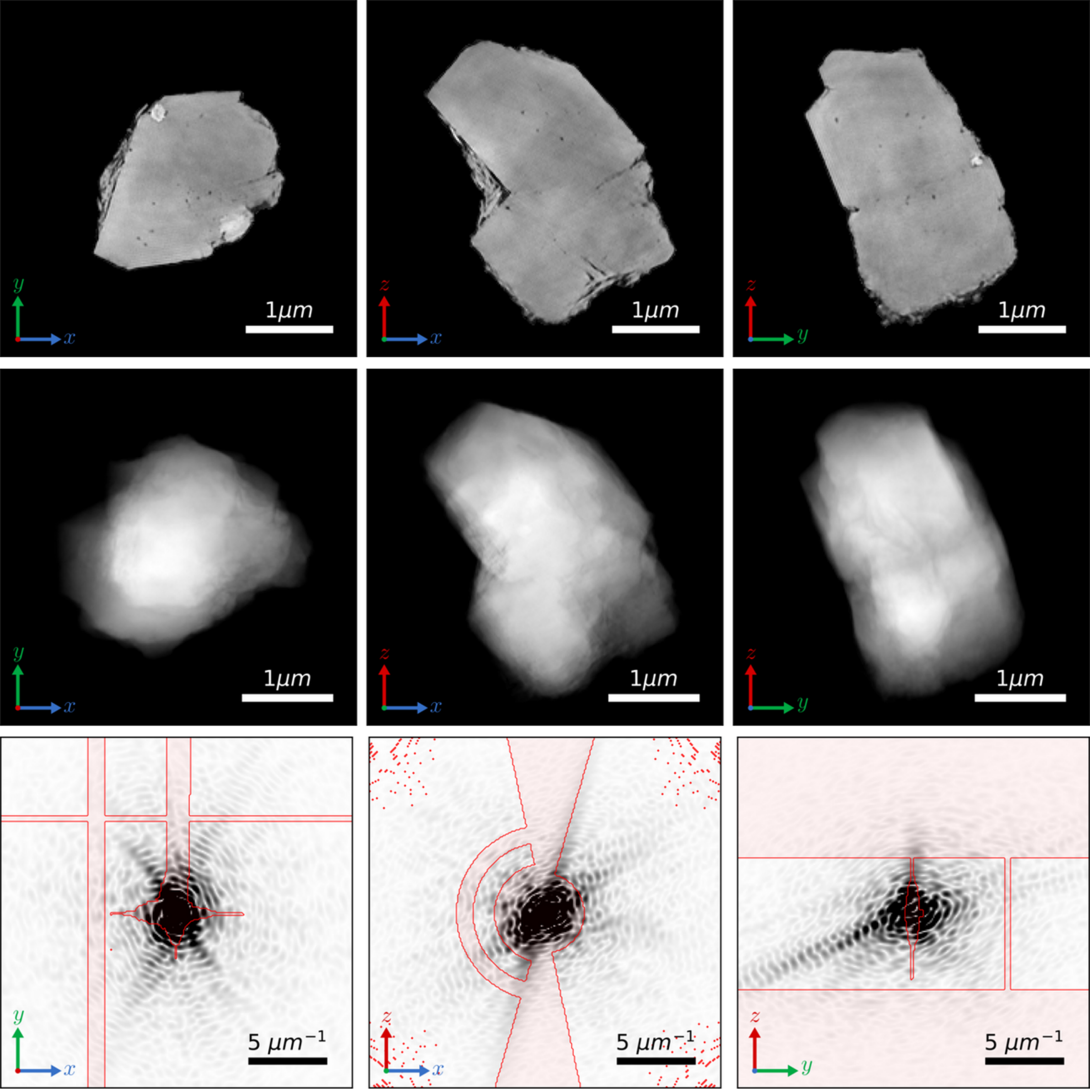
Three-dimensional reconstruction obtained with the TVRAAR–ERHIO algorithm. Top row: orthogonal slices through the 3D electron density. Middle row: orthogonal projections of the reconstruction. Bottom row: orthogonal slices through the reconstruction in Fourier space with the masked regions shaded red. The highest intensities have been clipped to help visualize the lower values. Note that a reversed gray-scale colormap is used in the bottom row so that the smallest values correspond to white. All images are shown on a linear scale.

**Figure 5:**
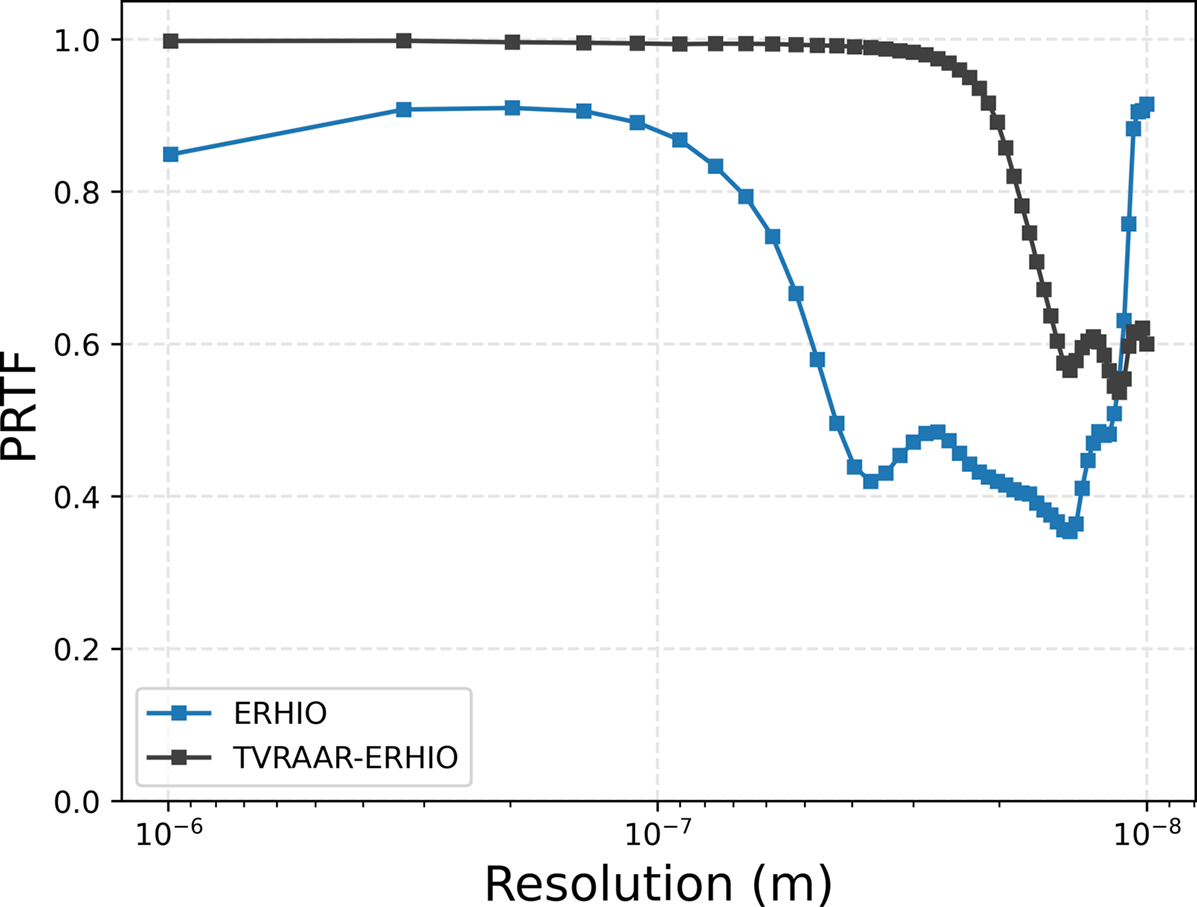
PRTF values for ERHIO and TVRAAR–ERHIO reconstructions corresponding to Figs. 3 ▸ and 4 ▸, respectively.

**Figure 6:**
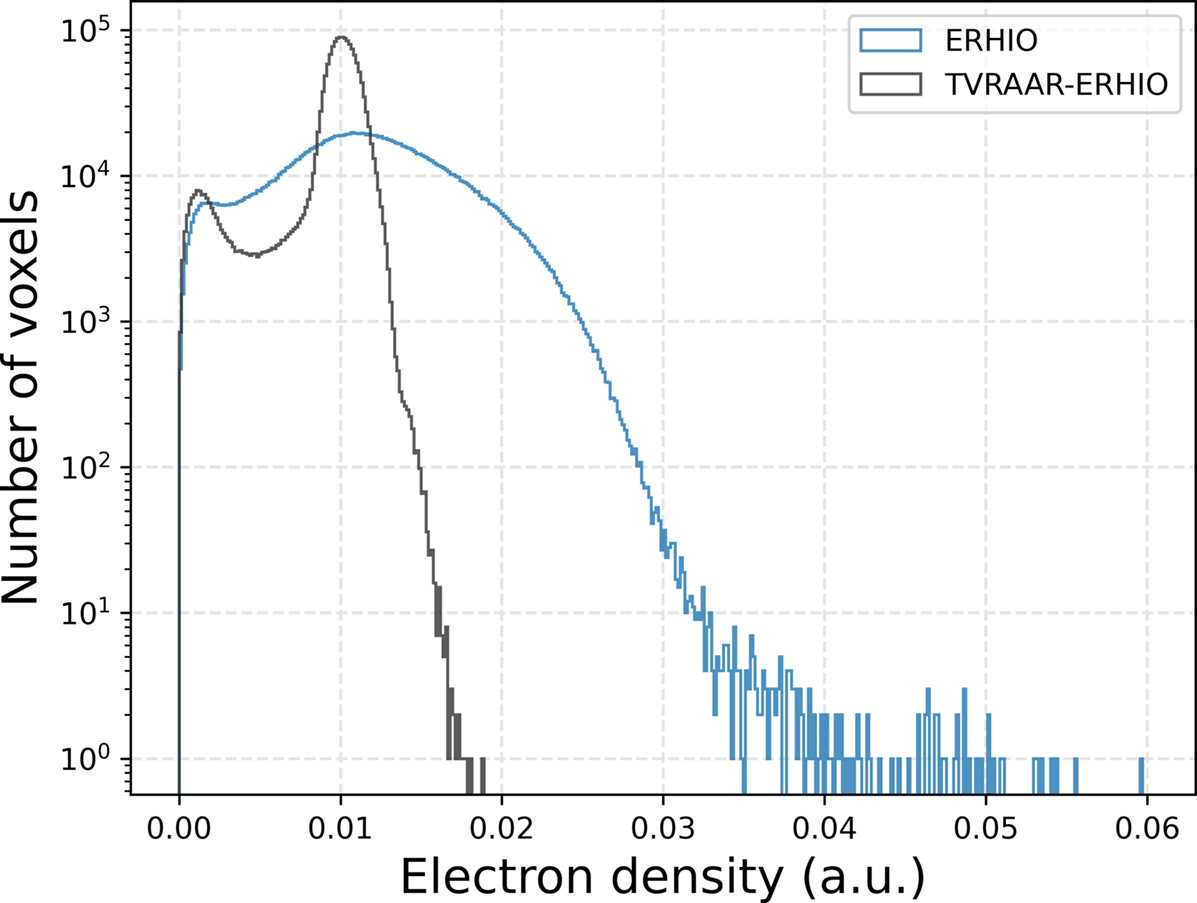
Plots of the electron density for the two reconstruction procedures.

Histograms of the electron densities from within the support are plotted in Fig. 6 ▸. The TVRAAR–ERHIO reconstruction shows a much more defined peak near 0.01 which is what we would expect from this sample. The low-frequency variations still present in the reconstruction, and visible in Fig. 4 ▸, limit the electron density resolution and the ability to discern between similar materials. In contrast, the rapid oscillations in the ERHIO reconstruction smear out the values across a much larger range which results in the broader profile.

Before concluding, it is important to describe the limitations of this approach. First, the unconstrained modes are truly unknown without any additional *a priori* knowledge and we do not claim that the recovered signal from TV refinement is the ‘correct’ one. However, in practice, reconstructions using TV refinement should be closer to the ground truth in the vast majority of cases. Secondly, when we compare reconstructions in Fourier space we see that the TV refinement recovers signal which is much closer to the surrounding measurements and to typical diffraction patterns. Therefore, our argument is that theoretically we are no more ‘correct’ but from a practical perspective we obtain reconstructions which are in-line with past experiences and surrounding measurements. It is also worth mentioning that the presence of the beamstop introduces low-frequency artifacts in the reconstructions which prevents the accurate determination of sample electron density. As our results demonstrate, the TV algorithms perform better in mitigating the artifacts, but do not eliminate them completely. Therefore, it is important to collect data with the smallest beamstop possible. Future work in mitigating artifacts could include replacing the TV cost function with another one which makes physical sense. Then, the reconstructions obtained with different cost functions could be compared to provide uncertainties on the reconstructions.

The authors believe that this approach is a general technique that can be applied to a wide range of samples. While it attempts to recover the minimum TV solution for unmeasured frequencies, the formulation does not place restrictions on the sparsity of the sample for the remaining frequencies. The algorithms should not produce the best solution if the sample has large oscillations at unmeasured frequencies. One possible scenario where this could occur is when a sample is illuminated by a vortex beam. Another scenario is when the sample has a rapidly oscillating electron density such as a very porous sample with insufficient sampling or resolution in real space. The high-frequency oscillations will likely limit the gradient descent step size and slow the convergence of the TV algorithm in recovering the unmeasured frequencies. However, in the majority of experiments this approach should improve the quality of the reconstructions.

In conclusion, approaches have been described and investigated for reducing artifacts in coherent diffraction imaging experiments where the dataset contains regions of missing data. Unlike most work that utilizes the TV cost function, the aim was not to regularize the solution but merely bring uniqueness back to the phase retrieval problem. This had the advantage that there is no need to set an arbitrary constant to balance the importance of data fidelity and TV terms. The algorithms have been demonstrated on a large (650^3^) 3D dataset and can be used for 2D and Bragg CDI datasets as well. We believe that the simplicity, applicability to large datasets, and improvements in reconstruction quality suggest that these algorithms should be applied to CDI datasets to minimize artifacts related to missing data.

## Supplementary Material

Figures S1 to S4. DOI: 10.1107/S1600577524010956/gui5001sup1.pdf

## Appendix A Phase retrieval algorithms

The specific details of the phase retrieval algorithm are provided below. It is worth noting that no positivity constraints were used in the phase retrieval procedures.

The ERHIO algorithm uses a combination of ER and HIO. We define an intermediate algorithm, ERHIO′ as ERHIO′ := ER_100_ (ER_10_HIO_20_)_5_ HIO_1000_ (ER_10_HIO_20_)_5_ HIO_1000_ ER_100_. The full ERHIO algorithm is given by: ERHIO := (ER_10_HIO_20_)_10_. Subscripts indicate the number of iterations. For example, ER_10_HIO_20_ means 20 iterations of HIO followed by 10 iterations of ER. The TV–ERHIO is defined as: TV–ERHIO := ER_10_ ERHIO′, and the TVRAAR–ERHIO algorithm is defined as: TVRAAR–ERHIO :=  ERHIO′. Superscripts on the TV and TVRAAR algorithms indicate the step size. Notice that the TV step size decreases as the algorithm progresses. The reason for this is that we aim to recover the lowest spatial frequencies with relatively large step sizes while trying to avoid regularization by lowering this value towards the end. A Python script which implements the TV refinement algorithm from Section 2.2.1 is provided in the following repository: https://bitbucket.org/malmy002/tvcdi.
