# Supplementary material for: Reduction of artifacts associated with missing data in coherent diffractive imaging
Source: J Synchrotron Radiat. 2025 Jan 1;32(Pt 1):210–6. doi: 10.1107/S1600577524010956 (PMC11708845; doi:10.1107/S1600577524010956)
Supplement: Supplementary file 1 [file s-32-00210-sup1.pdf]

Reduction of artifacts associated with missing data in coherent diffractive imaging
Supplementary information

The figures below show reconstructions from simulated datasets using the three different phase retrieval algorithms. The final reconstruction for each algorithm is an average of 10 independent reconstructions. The images show orthogonal slices through the 3D volume while the bottom row shows the true sample image used to simulate the datasets.

The reconstructions shown in Fig. S1 correspond to beamstop diameter, missing wedge angle, and SNR values of 8 speckles (20 pixels), 10 degrees, and 12 dB respectively. In Fig. S2, the beamstop diameter, missing wedge angle, and SNR were set to 4 speckles (10 pixels), 10 degrees, and 9.7 dB respectively. Figure S3. shows reconstructions associated with beamstop diameter, missing wedge angle, and SNR were set to 4 speckles (10 pixels), 60 degrees, and 18.8 dB respectively.

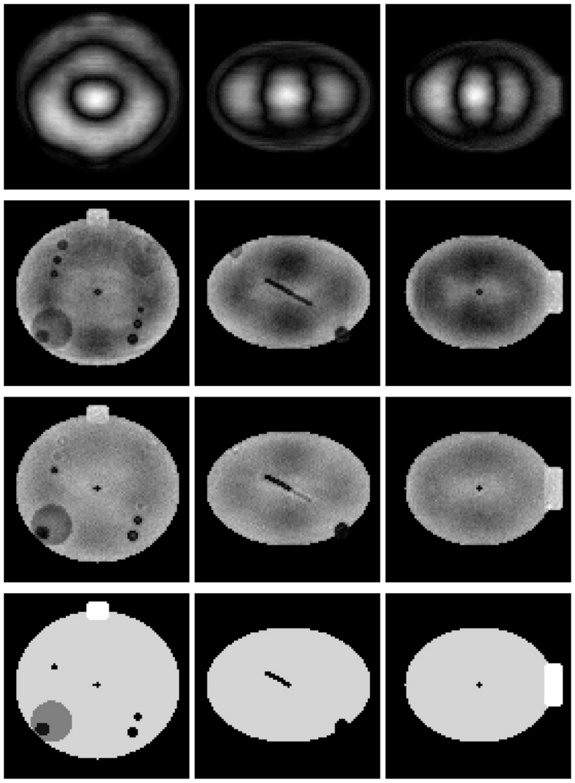

Figure S1: Slices through the 3D reconstruction recovered using (top row) ERHIO, (2nd row) TV-ERHIO and (3rd row) TVRAAR-ERHIO compared to the (bottom row) exact particle.

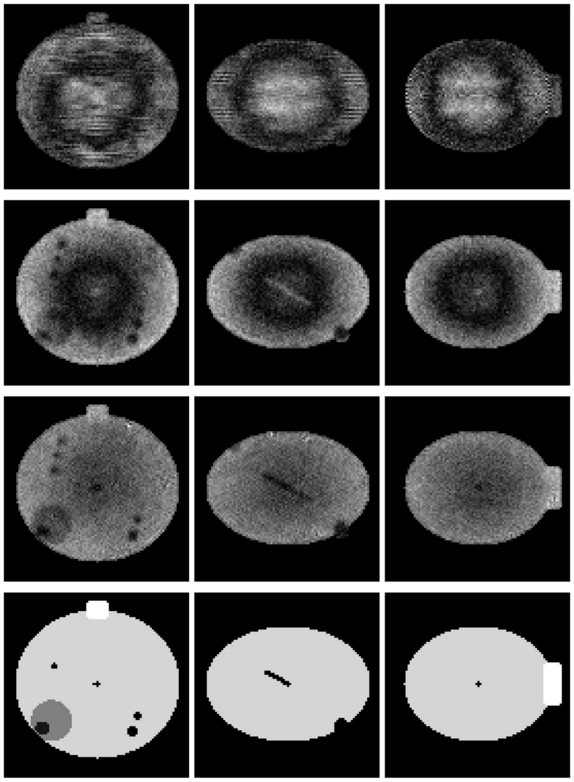

Figure S2: Slices through the 3D reconstruction recovered using (top row) ERHIO, (2nd row) TV-ERHIO and (3rd row) TVRAAR-ERHIO compared to the (bottom row) exact particle.

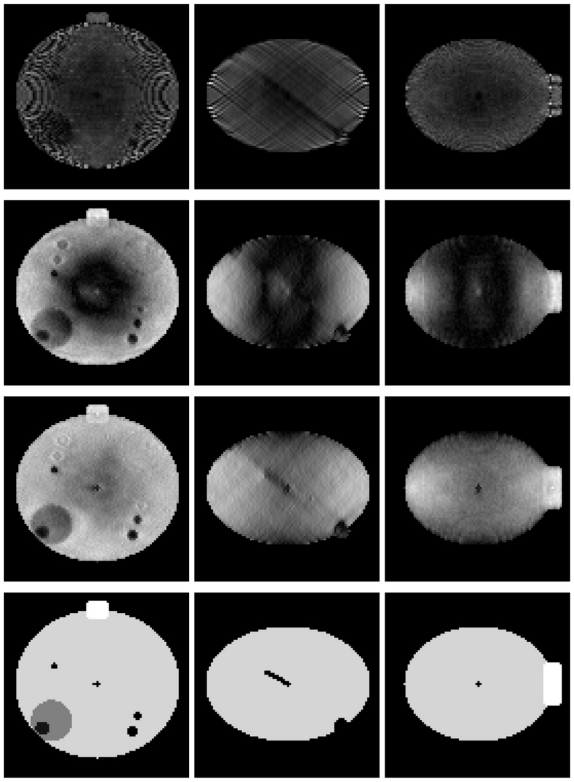

Figure S3: Slices through the 3D reconstruction recovered using (top row) ERHIO, (2nd row) TV-ERHIO and (3rd row) TVRAAR-ERHIO compared to the (bottom row) exact particle.

The plots in Fig. S4 show the evolution of the relative error for each of the three algorithms. Each line is the average value of the 10 independent reconstructions and the shaded regions indicate one standard deviation. The first three plots (a-c) correspond to the reconstructions shown in Figs. S1-S3 above respectively, while the last plot corresponds to the dataset with the largest SNR point from Fig. 2(b). All the algorithms begin with the same implementation of ERHIO and therefore have essentially the same error values at the beginning. Interestingly, for large missing wedge angles, as shown in Fig. S3 and Fig. S4(c), the error for TV-ERHIO grows with iteration number . This shows the inability to recover the low-frequency information. However, by inspecting the corresponding reconstruction in Fig. S3, we can see that some features have become more visible relative to the ERHIO reconstruction showing that there was still value in using TV-ERHIO. Figure S4(d) shows that under favorable conditions such as: large SNR, small missing wedge and beamstop, all three algorithms recover solutions which are close the true values. Clearly, the TVRAAR-ERHIO algorithm shows the best overall performance in terms of both the visual appearance and relative error. By inspecting the reconstruction algorithms, one will notice that the change in behavior of the error corresponds to a change in algorithm. Lastly, because the aim was to not incorporate regularization, we should expect that the noise level should place a lower bound on the lowest achievable error value. This is visible when comparing the plots in Fig. S4(a-c) to Fig. S4(d), which has the largest SNR.

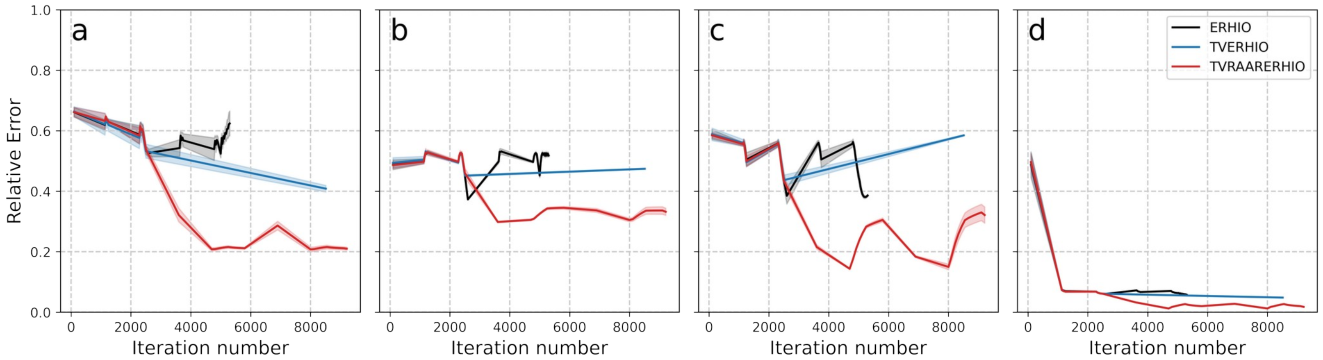

Figure S4: Relative error evolution for four datasets. (a-c) Relative errors corresponding to figures S1-S3 in the supplementary information. (d) Relative error associated with the highest SNR dataset shown in figure 2(b) in the main text. The lines correspond to the average of the 10 independent reconstructions and the shaded regions indicate one standard deviation.
